# High and low mutational burden tumors versus immunologically hot and cold tumors and response to immune checkpoint inhibitors

**DOI:** 10.1186/s40425-018-0479-7

**Published:** 2018-12-27

**Authors:** Saman Maleki Vareki

**Affiliations:** 1 Department of Oncology, University of Western Ontario, London, ON Canada; 2 Cancer Research Laboratory Program, Lawson Health Research Institute, London, Ontario Canada

**Keywords:** Immunotherapy, Cancer, Checkpoint inhibitors, Hot and cold tumors, T-cell-inflamed and non-T-cell-inflamed tumors, High TMB and low TMB tumors

## Abstract

Tumors responding to immune checkpoint inhibitors (ICIs) have a higher level of immune infiltrates and/or an Interferon (IFN) signature indicative of a T-cell-inflamed phenotype. Melanoma and lung cancer demonstrate high response rates to ICIs and are commonly referred to as “hot tumors”. These are in sharp contrast to tumors with low immune infiltrates called “cold tumors” or non-T-cell-inflamed cancers, such as those from the prostate and pancreas. Classification of tumors based on their immune phenotype can partially explain clinical response to ICIs. However, this model alone cannot fully explain the lack of response among many patients treated with ICIs.

Dichotomizing tumors based on their mutation profile into high tumor mutation burden (TMB) or low TMB, such as many childhood malignancies, can also, to some extent, explain the clinical response to immunotherapy. This model mainly focuses on a tumor’s genotype rather than its immune phenotype. High TMB tumors often have higher levels of neoantigens that can be recognized by the immune system. In the current era of immunotherapy, with the lack of definitive biomarkers, we need to evaluate tumors based on both their immune phenotype and genomic mutation profile to determine which patients have a higher likelihood of responding to treatment with ICIs.

## Main text

An “immunotherapy tsunami” has overtaken the field of oncology in recent years. Perhaps the most impressive effect of immunotherapy is the near-complete durable responses observed in a substantial fraction of patients with highly refractory and late-stage cancers treated with immune checkpoint inhibitors (ICIs). As one example, among advanced melanoma patients treated with ipilimumab (an anti-CTLA-4 antibody), some remain alive ten years after treatment with this drug [1]. Other ICIs targeting programmed cell death-1 (PD-1) and its ligand (PD-L1) have changed oncology practice in multiple disease sites and helped many patients. However, most cancer patients, across a spectrum of anatomical sites, do not respond to these drugs. For example, ovarian cancer has a modest somatic mutation burden and is somewhat infiltrated by T-cells; however, it displays a low response rate to various ICIs [2–4]. In addition, prostate and pancreatic cancers are both cold tumors with low tumor mutation burden (TMB) and are consequently not responsive to ICIs – a situation that creates a challenge for the successful application of immunotherapy in these cancers [4–6].

Currently, there are no definitive biomarkers to predict patient response to ICIs, nevertheless, and with all its limitations, PD-L1 expression on malignant and immune cells was identified early on as a biomarker of response to anti-PD-1/PD-L1 therapy [7–9]. Recently, TMB has been proposed as a biomarker of response to ICIs [10]. This is because a common feature among cancers with a higher probability of response to these drugs is the higher prevalence of somatic mutations in their genomes. For example, melanoma and non-small cell lung cancer (NSCLC) – both genomically unstable tumor types – are among those malignancies with higher overall responses to anti-PD-1 therapy. These cancers share a common feature of being UV- or carcinogen-induced with a high TMB in their genomes [11]. Most tumors can be categorized as either high TMB and more likely to respond to ICIs or low TMB with a low probability of response to ICIs. This classification of cancers is mainly based on their genotype and is rationally explainable, as high TMB tumors often have more neoantigens that could be recognized by processes involved in antitumor immunity, making such cancers more likely to respond to anti-PD-1/PD-L1 therapy. It is worth noting that anti-PD-1/PD-L1 antibodies commonly re-invigorate tumor-reactive T-cells, but do not induce their formation. High objective response rates (ORR) to ICIs in cancers with microsatellite instability or mismatch repair (MMR) deficiency is a prime example of high TMB tumors responding to immunotherapy with ICIs. MMR deficiency induces frameshift mutations in tumors that can increase the likelihood of neoantigen formation in tumors [12]. Due to the accumulation of neoantigens and presence of more tumor-reactive T-cells in the tumor microenvironment, MMR-deficient tumors are most likely to be associated with high ORR to ICIs. This is the basis for the first U.S. Food and Drug Administration (FDA) tissue-agnostic approval of pembrolizumab (an anti-PD-1 antibody) for the treatment of adult and pediatric cancers with unresectable or metastatic microsatellite instability-high or MMR-deficient solid tumors.

Defining tumors as high TMB and low TMB – based on the prevalence of somatic mutations in their genome – can be used, to some extent, to differentiate between cancers with higher and lower probability of response to ICIs. However, this model does not provide a universal definition of which patient will respond to these drugs and which will not. For instance, the majority of melanoma or NSCLC patients do not respond to these drugs despite having high TMB. Moreover, some patients with renal cell carcinoma (RCC), which is a cancer with low TMB, respond to ICIs, hence the FDA approval of nivolumab (an anti-PD-1 antibody) for the treatment of patients with metastatic RCC. Although a high prevalence of insertions and deletions (indels) can result in the formation of some neoantigens, RCC still has a significantly lower burden of somatic mutations than melanoma and NSCLC (true high TMB tumors) [13]. Another example of this phenomenon is the high ORR among Merkel Cell Carcinoma (MCC) patients with Merkel cell polyomavirus (MCPyV) positive tumors in response to treatment with avelumab (an anti-PD-L1 antibody) despite the relatively low mutation burden in this tumor type [14]. Interestingly 20% of MCC tumors are negative for MCPyV and are caused by chronic UV exposure hence have high TMB in their genome. However, the MCPyV positive-MCC tumors with low TMB have equivalent or higher response rates to ICIs than the MCPyV-negative MCC tumors [15]. HPV^+^ head and neck squamous cell carcinomas (HNSCC) have mutation loads comparable to those in HPV^−^ HNSCC, but patients with HPV^+^ HNSCC have better outcomes and slightly better responses to ICIs. Interestingly, both MCC and HPV^+^ HNSCC are virally-induced cancers with higher T-cell infiltration in response to the viral antigen – an observation that may explain their improved ORR to ICIs and better patient outcome. Viral antigens, similar to most neoantigens, are foreign to the immune system providing additional targets for T-cells; however, these antigens do not contribute to a tumor’s high TMB status [16].

Indeed, the T-cell-inflamed or “*hot”* tumor phenotype has been proposed as a predictive model of response to ICIs [17]. In this model, cancers are differentiated by expression of T-cell markers and interferon (IFN) signature, regardless of their mutational status. As such, this model mainly characterizes a tumor’s immune phenotype rather than its mutation burden (genotype). This strategy of tumor stratification can explain why some patients with tumors expressing immunosuppressive molecules such as PD-L1 and/or indoleamine 2,3-dioxygenase (IDO) are more likely to respond to ICIs compared to tumors lacking these molecules. This is mainly because the expression of these immunoregulatory molecules is secondary to the presence of antitumor T-cells and immune effector cytokines such as IFN-γ [17]. Therefore, other than an oncogene-mediated expression of PD-L1 or IDO, the presence of such immunosuppressive molecules points to the presence of a suppressed pre-existing antitumor immunity that could be re-invigorated by anti PD-1/PD-L1 immunotherapy.

We recently discovered that HPV^+^ HNSCC patients with a distinct signature of T-cell exhaustion markers, indicative of a T-cell-inflamed or hot tumor phenotype, have much higher survival rate than HPV^+^ HNSCC patients that lack such T-cell-inflamed phenotype [18]. This observation could suggest a mechanistic explanation as to why patients with the same type of cancer do not respond similarly to the same treatment. Moreover, a melanoma tumor with β-catenin activation is a high TMB tumor with a T-cell-excluded phenotype that is not likely to respond to ICIs because of active T-cell exclusion from the tumor parenchyma and retention of these cells at the stroma of the tumor, which effectively transforms a high TMB tumor into an immunologically cold tumor [19, 20]. As opposed to immune-excluded phenotype, the immune –desert phenotype is often characterized by the lack of T-cell presence in the tumor parenchyma or stroma [20]. This phenotype is also non-responsive to ICIs and is classified as a non-T-cell-inflamed subtype [20].

**Fig. 1:**
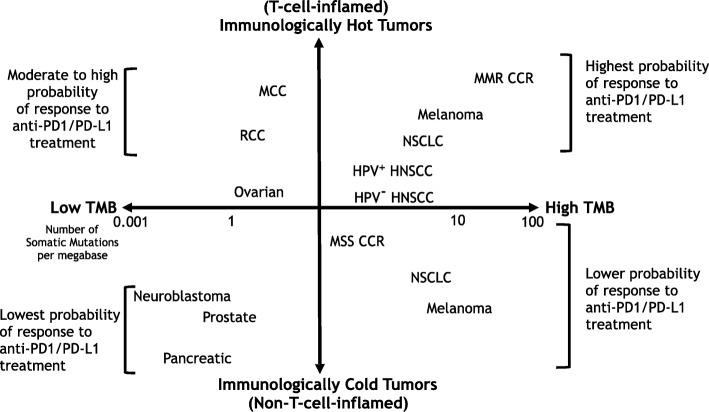
Schematic diagram of high TMB and low TMB tumors versus hot and cold tumor types and their likelihood of response to ICIs. Tumors with higher somatic mutation prevalence such as melanoma and NSCLC are among high TMB tumors (highly mutated). However, some high TMB tumors can be either hot (T-cell-inflamed) or cold (non-T-cell-inflamed) based on their T-cell and IFN signature (immune phenotype). MMR-deficient CCR cancers are both high TMB and immunologically hot, while MSS CCRs have relatively high mutation rate, but non-T-cell-inflamed. HPV^+^ HNSCC are similar to HPV^−^ HNSCC in their mutation burden, but are more T-cell-inflamed. MCC, ovarian cancer, and RCC have modest mutation rates with a relatively T-cell-inflamed phenotype. Neuroblastoma, prostate, and pancreatic cancers are both immunologically cold and have relatively low mutation burden

It is crucial to understand that although a tumor’s genotype can shape its microenvironment, higher mutation rates are not necessarily equal to higher immune infiltration into the tumor [21]. Thus, a high TMB tumor doesn’t always have an immunologically hot (T-cell-inflamed) phenotype. Non-T-cell-inflamed (immunologically cold) microsatellite stable (MSS) colorectal cancers with relatively high TMB are also non-responsive to ICIs. This could be partially explained by the recent report that TGFβ plays a crucial role in excluding T-cells from the tumor microenvironment [22]. Taken together, the high/low TMB and immunologically hot/cold models can, in combination, help explain some current clinical observations of immunotherapy response (Fig. 1). Unfortunately, the differences between these models are often overlooked. However, effective use of immunotherapy in the era of precision medicine requires understanding of both the genotype of the tumor and its immune phenotype. Nevertheless, high TMB and immune signature of tumors can be used as independent biomarkers for patient selection for treatment with ICIs in at least some tumor types. They can also guide us in designing scientific and clinical combination studies that may succeed in transforming ICI non-responsive cancers into those responsive to ICIs. For example, a cancer such as metastatic melanoma, which has high TMB, but is non-T-cell-inflamed (genomically high levels of mutations but phenotypically an immunologically cold tumor), can be rendered responsive to ICIs by treatment with a combination of oncolytic virus therapy and anti-PD-1 therapy [23]. Viral infection can induce a T-cell-inflamed phenotype in injected tumors that can be re-invigorated and sustained with anti-PD-1 therapy. Importantly, the high TMB nature of melanoma and the presence of neoantigens in distant metastatic lesions allows the now-boosted antitumor immunity to target and destroy those lesions. The same strategy may not be as effective in tumors with low levels of somatic mutations, where fewer neoantigens exist (in prostate and pancreatic cancers, for example). However, strategies to transform tumors with low TMB into high TMB tumors by inducing MMR-deficiency have been proposed [12]. These strategies, in combination with methods that can induce T-cell-inflamed phenotypes such as combination treatment with oncolytic viral therapy, have the potential to render a large group of cancers responsive to ICIs.

The two models of TMB and immune signature (T-cell-inflamed versus non-T-cell-inflamed) should be kept in mind during the design of preclinical (discovery) and early clinical studies of immunotherapeutic combinations. Given the enthusiasm for precision medicine in oncology, we can assume that the probability of success of immunotherapy in cancer patients can be increased by testing patient’s tumors for their mutation and immune profiles, which would allow for the optimal design of a personalized treatment regime. For example, a NSCLC patient with a high TMB but immunologically cold (non-T-cell-inflamed) cancer could likely benefit from anti-PD-1 immunotherapy in combination with another agent or treatment modality, such as radiation or chemotherapy, which can help transform the tumor microenvironment to T-cell-inflamed (immunologically hot) rendering it responsive to treatment with an anti-PD-1 drug [24, 25]. Although finding predictive biomarkers for immunotherapy is an area of active investigation, these two models and their distinctions need to be kept in mind, not only for discovery research but also for treatment strategies that involve a combination of anti-PD-1/PD-L1 drugs and other immunotherapeutics and non-immunotherapeutics agents. The recent failure of the phase III combination of pembrolizumab and epacadostat (an IDO inhibitor) in melanoma patients demonstrates the urgent need for the development of better biomarkers and patient selection for these combination studies [26]. Ultimately, we should use the information on patient’s tumor genotype (mutation burden) and its immune profile to decide which combination of currently approved drugs or agents in development is best for the patient. This strategy along with advances in precision medicine and the use of next generation sequencing in molecular profiling of cancer will lead us to the era of personalized cancer immunotherapy, in which tapping into the power of the immune system will be coupled with the rest of our arsenal against cancer to ensure durable remissions are achieved in hard-to-treat cancers.
